# Gynecology and Obstetrics

**Published:** 1894-03

**Authors:** 


### GYNECOLOGY AND OBSTETRICS.

EDITED BY DR. VIRGIL O. HARDON.

### ON THE USE OF THE CURETTE.

Curetting (says Fancourt Barnes, M. D., F. R. S., Edin.) finds its chief application in the various morbid conditions of the uterus which result from the imperfect evacuations of its contents, after a full time labor or an abortion. . . . . . Among the conditions not directly associated with pregnancy, follicular endometritis very often can only be cured by the use of the curette; in this condition, fungous excrescences of the mucosa may be removed from the uterus, which in themselves constitute an obvious cause of the symptoms from which it is sought to relieve the patient. In the hands of some gynecologists, curetting is undertaken in cases of malignant disease of the uterus which do not admit of a radical operation; the procedure removes considerable masses of breaking-down malignant growth, and by freeing the patient to some extent from the risks of septic abortion, improves her general condition. Lastly, in doubtful cases, curetting may be undertaken for purposes of diagnosis; the nature of an intra-cervical growth being uncertain, a fragment is removed by the curette for microscopic examination. . . . . . It is to be clearly understood from the outset that curetting, being a severe operation, attended by certain grave risks, is not to be lightly undertaken in any case. The operative technique is simple, and it is therefore likely to be looked upon as a trifling affair which may, without fear, be undertaken by the unskillful. This view is strongly to be deprecated, for from time to time serious complications arise during or after the operation, which call for all the recources of skill and experience.

If the operation of curetting the uterus is to be properly and safely performed, it is essential that the cervix be sufficiently dilated to admit the entrance of the index finger of the operator. Except, therefore, in early post-partum cases, when the cervix has not yet contracted, preliminary dilatation becomes necessary. There are two methods of performing dilatation of the cervix, the rapid and the gradual. Rapid dilatation is performed as the first step in the operation, either by the successive introduction of Hegar's graduated dilators until a size has been passed which allows the operator's finger to follow, or by the use of the metal dilators of Sims or Ellinger. Gradual dilatation is performed by the use of tents, and requires upwards of twelve hours for its completion; it is, therefore, commenced the night before the operation by the introduction of as large a size of tent as the cervical canal will admit without the employment of force; when in the course of a few hours the tent becomes loose owing to some amount of dilatation having occurred, it is removed and a larger size substituted. Of the two methods of dilatation the gradual is to be preferred; it is in the first place a more scientific method to employ a gradually acting force, and the result is obtained with less mechanical injury to the tissue of the cervix. If the cervix be markedly indurated, the rapid method is very apt to cause considerable laceration, which may need repair after the curetting is completed. Gradual dilatation is, moreover, accompanied by such softening of the tissues of the cervix, that subsequent manipulations are rendered much more easy. Some operators combine the two methods, and commencing the dilatation with a tent, complete it with the rapid process.

Sponge tents emphatically condemned and those of Laminaria (seatangle) as strongly recommended, not less than four inches long, and employed under rigid antisepsis. There are two chief kinds of curette, the sharp and the blunt. Simon's spoon is preferred among sharp curettes, and the dull wire of Gaillard Thomas among blunt instruments.

There are certain definite risks to be avoided, perforation, troublesome hemorrhage and inflammatory troubles, all of which can be either entirely prevented, or their danger lessened by appropriate precautions on the part of the operator.—*Medical Week-Progress.*

---

### THE EFFECT OF CASTRATION ON WOMAN.

Dr. Goodell (*Medical News*) says:

My reasons for conservative treatment are, that the complete extirpation of these organs tends to destroy the sexual feeling, to disturb the mental equilibrium, and to produce prolonged nervous perturbations, all of which come from the abrupt and untimely suspension of menstruation. There is yet another very excellent reason for this advice: The majority of physicians, and all laymen, look upon women deprived of their ovaries as unsexed. Just as castration is in the male, so the analogous operation is in the female deemed a sexual mutilation to which common consent attaches a stigma. No woman would marry a eunuch, and few men would wed a woman deprived of her ovaries. In my own practice I have known of several very sad cases of marriage engagements broken off, of marital infidelities, and of bitter estrangement between husband and wife, all of which would have been avoided had one ovary been spared, or, indeed, had a mere fragment of one been left behind.

Upon the question of the removal of the uterine appendages for the cure of insanity and of epilepsy, I have very few words to say, but they are all based upon cases occurring in my own practice. If the insanity is limited to periodic outbreaks strictly ovarian in their character, and with the menstrual flux as a storm-center; if the epileptic fits are preceded by an ovarian aura—that is to say, if they pivot around the monthly period and appear at no other time—the removal of the appendages, by suppressing a pernicious menstruation, usually will bring about a cure in either disease. But when these organs are extirpated merely as a panacea *per se* for these mental and neural disorders, irrespective of an ovarian origin, the operation affords no relief. At the same time I am free to confess that, in order to stamp out insanity, I am strongly inclined to advocate the legal castration of every man and of every woman who is the unfortunate victim of this hereditary curse.—*Am. Lancet.*

---

### THE TREATMENT OF ECLAMPSIA.

From a new study of this important subject Dr. Charpentier, of Paris, has been led to draw the following conclusions: 1. As every albuminuric pregnant woman is liable to eclampsia, and as milk diet gives such marvelous results in albuminuria, and especially in the albuminuria of pregnancy, one should examine with the greatest care the urine of pregnant women; and if albumin be found in no matter how small a quantity, milk diet, absolute and exclusive, should be instituted at once. This is the best prophylactic treatment of eclampsia. 2. Given a case of eclampsia. If the patient is strong and vigorous and much cyanosed, begin by bleeding to the extent of 400 or 500 grams. Then administer chloral, at the same time getting the patient to begin on milk as soon as possible. 3. If the patient is delicate, and the cyanosis less marked and the attacks less frequent, the treatment should be limited to chloral. 4. Wait for labor to come on of itself, and allow it to terminate spontaneously, whenever possible. 5. Cases of tardy labor due to uterine atony should be terminated by the forceps or version, if the child be still living; or by cephalotripsy, basiotripsy, or cranioclasis, if dead. 6. Wait before any such interference until the maternal parts are in such condition that it can be done without violence, and therefore without danger to the mother, namely, until complete dilatation of the cervix. 7. Reserve induced labor for the exceptional cases in which treatment by drugs has wholly failed. 8. Cæsarean section, accouchement force, and above all, accouchement force with deep incisions of the cervix, are to be rejected absolutely.—*Archives de Tocologie.*—*Medical Review.*

---

### CONCEPTION DURING THE PUERPERAL PERIOD.

Dr. Brasseur relates the case of a woman twenty-two years of age, who was delivered on July 4, 1892, of her first child. July 8th she practiced coitus, and was again delivered March 10, 1893, of a healthy child. Calculating from the date of coitus, the second pregnancy lasted two hundred and forty-three days, that is, twenty-seven days less than normal. This case has caused considerable discussion. Ovulation must have existed in the woman on the fourth day after the delivery, and it was necessarily quite independent of menstruation. Dr. Koenig, who actually observed the case, draws from it the following deductions: 1. A gestation period of two hundred and forty-three days after a fecundaitng coitus may produce a viable child. 2. The spermatozoa can live in the lochial secretions. 3. The functional activity of the ovaries is not completely suspended during pregnancy. The Graafian follicles so open that they may burst a very short time after delivery. 4. Ovulation and menstruation may occur independent of each other. 5. Among vigorous women, during the period immediately following confinement the uterine mucous membrane may undergo a rapid regeneration which renders possible the implantation of a fecundated ovule immediately after delivery.—*Gazette Médicale de Léige.*

---

### TO PREVENT CRACKED NIPPLES.

Dr. Virginia M. Davis, of New York, is accustomed to apply lanolin with the onset of labor four times daily till lactation is established. The nipples are then, after each nursing, annointed with the following:

℞. Tr. benzoin comp . . . . . . . . . . . . . . . . . . gtt. xv.

Ol. olivæ . . . . . . . . . . . . . . . . . . . . . . f drams lj.

Lanolin . . . . . . . . . . . . . . . . . . . . . . drams vj.

M. ft. ungt.

—*Prescription.*

